# From Adaptive Resilience to Catastrophic Systems Collapse: Endothelial Entropy, Ferroptotic Propagation, and the Maternal Point of No Return in Emergency Peripartum Hysterectomy

**DOI:** 10.3390/ijms27146484

**Published:** 2026-07-21

**Authors:** Elena-Evelina Stoica, Stefan Oprea, Dan Dumitrescu, Adrian Vasile Dumitru, Matei Șerban, Răzvan-Adrian Covache-Busuioc, Corneliu Toader, Monica-Mihaela Cirstoiu

**Affiliations:** 1 Doctoral School, “Carol Davila” University of Medicine and Pharmacy, 050474 Bucharest, Romania; elena-evelina.stoica1025@rez.umfcd.ro (E.-E.S.); monica.cirstoiu@umfcd.ro (M.-M.C.); 2 Department of Obstetrics and Gynaecology, “Carol Davila” University of Medicine and Pharmacy, 050474 Bucharest, Romania; 3 Faculty of General Medicine, “Carol Davila” University of Medicine and Pharmacy, 050474 Bucharest, Romania; adriandumitru@umfro.com (A.V.D.); mateiserban@innbn.com (M.Ș.); razvancovache@innbn.com (R.-A.C.-B.); corneliutoader@innbn.com (C.T.); 4 Department of Anatomy, “Carol Davila” University of Medicine and Pharmacy, 050474 Bucharest, Romania; 5 Department of Pathology, Faculty of Medicine, “Carol Davila” University of Medicine and Pharmacy, 030167 Bucharest, Romania; 6 Puls Med Association, 051885 Bucharest, Romania; 7 Department of Neurosurgery, “Carol Davila” University of Medicine and Pharmacy, 050474 Bucharest, Romania; 8 Department of Vascular Neurosurgery, National Institute of Neurology and Neurovascular Diseases, 077160 Bucharest, Romania

**Keywords:** emergency peripartum hysterectomy, placenta accreta spectrum disorders, catastrophic postpartum hemorrhage, endothelial glycocalyx dysfunction, ferroptosis-mediated oxidative injury, mitochondrial bioenergetic failure, immunothrombotic microcirculatory collapse, precision computational obstetrics

## Abstract

Beginning with a general understanding of catastrophic obstetric collapse (COC), it has been established that a catastrophic obstetric collapse is typically the result of sudden massive bleeding requiring emergency peripartum hysterectomy (EPH); this is different from historical views of what constitutes a catastrophic obstetric collapse. Current studies have found evidence that a catastrophic obstetric collapse can be the result of a longer-duration process involving gradual maternal physiological destabilization, the culmination of which creates a “maternal point of no return” for the mother. As a result of disrupting the maternal–fetal interface in placenta accreta spectrum disorders (PASDs), there are many abnormalities present in the decidua, such as: defective decidualization, fragmentation of the extracellular matrix, aberrant angiogenesis, continued hypoxic signals, and the persistence of invasive trophoblastic phenotypes. These structurally fragile vascular interfaces will eventually undergo endothelial dysfunction, oscillatory shear stress, glycocalyx injury, oxidative damage and progressive depletion of the maternal vascular adaptive reserve. Chronic inflammation will also continue to amplify immune thrombosis, alter complement function, facilitate NETosis, and cause widespread instability in diffuse microvasculature, leading to a reduced ability of the maternal system to tolerate physiological stress while maintaining macrocirculatory stability. Additionally, invasive placentation may lead to mitochondrial dysfunction, decreased oxidative phosphorylation, disrupted intracellular calcium homeostasis, ferroptotic lipid peroxidation, and redox-mediated endothelial injury, leading to a progressive limitation in the mother’s bioenergetic adaptability to hemorrhage. Ultimately, these events seem to culminate in a threshold condition where endothelial disorganization exists along with capillary transit time heterogeneity, impaired oxygen diffusion, metabolic instability, and progressive desynchrony of vascular, inflammatory, coagulative and mitochondrial networks before eventual hemodynamic collapse. Therefore, based on these findings, we propose the concept of the “Maternal Point of No Return” as a transitional state in which physiological adaptations begin to fail and irreversibly destabilize at a systems level. Lastly, we review potential applications of current technological advancements, including artificial intelligence (AI), radiomic-based placental phenotyping, exosomal biology, physiological variability analysis, spatial multi-omics, and digital twin physiology, to enable future precision-obstetrics strategies to identify a decline in maternal resilience prior to irreversible decompensation.

## 1. Introduction

Pregnancy represents one of the most complex adaptive conditions in human physiology. Maintaining maternal–fetal homeostasis involves significant adaptive changes across maternal and fetal circulatory, endothelial, immune, endocrine, metabolic, coagulative, mitochondrial, and placental systems [1].

While each system has distinct functions, these systems operate within a complex multilevel network in which molecular signaling, cellular metabolism, tissue-level vascular remodeling, and systemic circulation interact and influence each other [2]. Thus, maintaining maternal stability depends not only on organ function, but also on preserving adequate adaptive reserve and facilitating communication between highly interconnected vascular, inflammatory, metabolic, and hemostatic systems [3].

Emergency peripartum hysterectomy (EPH) is considered one of the most aggressive interventions in obstetrics. Although numerous advances in antenatal imaging, obstetric anesthesia, transfusion medicine, interventional radiology, damage-control surgery, and intensive care unit (ICU) care have occurred since the first descriptions of EPH, EPH is still required when conservative treatment options fail to control life-threatening obstetric hemorrhage [4].

Indications for EPH can vary widely; however, the primary indications include placenta accreta spectrum (PAS), uncontrollable postpartum hemorrhage (PPH), uterine atony refractory to treatment, uterine rupture, severe placental abruption, and disseminated intravascular coagulopathy (DIC) [5]. The increase in global cesarean delivery rates and the subsequent rise in PAS have significantly increased the relevance of EPH, particularly in patients with previous uterine surgery, placenta previa, or antenatally suspected invasive placentation [6]. Presently, the standard approach for managing patients at high risk for EPH is based on established clinical guidelines. These include early recognition of PAS and other hemorrhage risk factors, referral to experienced multidisciplinary centers for evaluation and management, planned delivery when possible, blood product availability, use of cell salvage where appropriate, selective interventional radiology procedures, and rapid escalation to emergency hysterectomy for definitive hemorrhage control when maternal survival requires it [7]. While adherence to these guidelines has improved outcomes for patients at high risk for EPH, variability still exists. Some patients can tolerate extensive placental invasion or significant hemorrhage without loss of macrohemodynamic stability, whereas other patients experience catastrophic systemic collapse despite apparently comparable clinical scenarios [8]. Such variability suggests that blood loss and/or anatomic placental invasion alone may not provide sufficient explanations for catastrophic maternal collapse [9]. A systems-biology perspective may provide insight into why apparently similar clinical insults produce different maternal trajectories. Established literature supports the importance of endothelial dysfunction, inflammation, coagulopathy, and mitochondrial stress as contributing factors in critical illness and hemorrhagic shock; however, their relative contributions to catastrophic obstetric collapse and EPH have not been clearly elucidated [10]. Consequently, in this review, these mechanisms are discussed within a hypothesis-generating framework rather than as fully validated causal pathways in obstetric patients [11].

Normal pregnancy is characterized by profound cardiovascular adaptations, including plasma volume expansion, increased cardiac output, altered venous capacitance, endothelial vasodilation, angiogenesis, and redistribution of regional perfusion [12].

These adaptations depend on nitric oxide signaling, prostacyclin activity, endothelin regulation, renin–angiotensin signaling, sympathetic modulation, endothelial mechanotransduction, and recalibration of endocrine–metabolic processes [13]. In addition to these cardiovascular adaptations, maternal immune tolerance also depends on remodeling of decidual immunity through regulatory T-cell expansion, specialized uterine natural killer-cell activity, macrophage polarization, cytokine modulation, and trophoblast-mediated immunoregulation [14]. Additionally, hemostasis shifts toward a more procoagulant state during normal pregnancy, with increased fibrinogen levels, enhanced thrombin generation, altered platelet function, endothelial tissue-factor expression, and reduced fibrinolytic activity [15,16].

These physiological changes usually represent protective processes. However, under extreme obstetric stress, the same processes may become vulnerable sites of destabilization. Endothelial injury, glycocalyx degradation, impaired nitric oxide signaling, regional hypoxia, abnormal oxygen extraction, capillary derecruitment, and mitochondrial stress have all been identified as potential contributors to critical illness and may also participate in catastrophic obstetric deterioration due to severe hemorrhage or invasive placentation [17,18,19].

Coagulation has recently been recognized as an integrated immunovascular network that includes endothelial activation, platelet signaling, complement cascades, fibrinolytic regulation, tissue-factor signaling, and neutrophil extracellular trap formation [20]. In extreme obstetric stress, adaptive hemostasis can shift toward thrombo-inflammatory amplification through endothelial activation, platelet dysfunction, dysregulated fibrinolysis, complement activation, NETosis, and microvascular instability [21].

Immunometabolism and mitochondrial biology may provide additional perspectives on how these processes contribute to catastrophic obstetric collapse. Hemorrhage, ischemia–reperfusion injury, oxidative membrane damage, glutathione depletion, calcium imbalance, ferroptosis-related lipid peroxidation, and mitochondrial permeability transition can impair oxidative phosphorylation and reduce cellular adaptive capacity [22]. Since mitochondria regulate endothelial signaling, redox balance, innate immune activation, inflammasome signaling, and cell-death pathways, mitochondrial dysfunction could reasonably contribute to the transition from compensated instability to overt systemic failure [23].

However, the specific roles that these pathways play in catastrophic obstetric collapse remain emerging areas requiring clinical validation. Concepts derived from nonlinear physiology and critical-transition theory provide useful paradigms for organizing these observations. Complex biological systems approaching collapse may display increasing variability after perturbations, delayed recovery, rising autocorrelation, oscillatory instability, and loss of adaptive flexibility before overt failure becomes clinically evident [24]. Similar patterns have been documented in sepsis, cardiovascular instability, neurological disease, and ecological collapse [25]. By analogy, catastrophic obstetric collapse may involve a gradual reduction in maternal resilience before abrupt decompensation. The concept of a “maternal point of no return” is used throughout this review to describe the proposed transitional state between compensation and irreversible collapse. This concept should not be interpreted as a clinically validated threshold, but rather as a conceptual model explaining how cumulative disturbances of endothelial, immune, coagulative, metabolic, mitochondrial, and microvascular origin may converge toward irreversible maternal collapse [26].

Emerging technologies may eventually allow testing of this model. Artificial intelligence-based predictive analytics, radiomics analysis, Doppler-derived assessment of maternal circulatory parameters, laboratory-based temporal trajectory analysis, inflammatory biomarker assessment, physiological variability analysis, spatial transcriptomics, and single-cell technologies could help identify hidden patterns of instability prior to overt decompensation [27,28].

At present, however, these tools remain investigational for evaluating EPH and should be viewed as future research tools rather than immediate alternatives to current obstetric decision-making models. Therefore, this review presents a systems-biology overview of emergency peripartum hysterectomy using the conceptual model of the maternal point of no return. The review synthesizes established clinical knowledge regarding EPH and PAS with recent findings from vascular biology, endothelial mechanobiology, immunometabolism, coagulation science, mitochondrial physiology, ferroptotic signaling, nonlinear systems theory, and computational medicine. In doing so, it aims to provide a structured hypothesis for how invasive placentation and catastrophic hemorrhage may progressively reduce maternal adaptive reserve before overt decompensation becomes clinically apparent.

## 2. Placental Invasion Biology and the Progressive Destabilization of Maternal–Fetal Boundary Integrity

### 2.1. Scar Niche Pathobiology, Decidual Failure, and the Persistence of Invasive Trophoblast States

Placental development during normal pregnancy represents a tightly regulated process of invasion. Extravillous trophoblast cells develop an invasive phenotype for a limited duration that enables them to invade the decidua and remodel the spiral arteries. The process relies on spatial regulation, metabolic adaptation, immune surveillance, angiogenic signaling, and mechanotransductive factors. As such, the maternal–fetal interface may be considered a complex regulatory system that facilitates controlled trophoblast invasion while maintaining the structural integrity of the decidua and myometrium [29]. Therefore, the maternal–fetal interface can be viewed as a dynamic regulatory ecosystem that permits invasion of trophoblast cells into the decidua while preserving structural integrity [30].

This delicate equilibrium is progressively compromised at multiple molecular, cellular, inflammatory, and biomechanical levels in placenta accreta spectrum (PAS). The clinical implications of PAS include impaired normal placental separation, increased risk of severe postpartum bleeding, and the need for planned or emergency hysterectomy when conservative alternatives are unsafe or ineffective. At a biological level, the maternal–fetal interface gradually loses part of its regulatory capacity and develops characteristics of increasing invasion permissiveness. These characteristics include impaired decidual resistance, breakdown of the extracellular matrix (ECM), inflammatory and angiogenic tissue remodeling, and persistent activation of trophoblastic cells [31].

The area of contact between mother and fetus during pregnancy is known as the maternal–fetal interface. The maternal–fetal interface includes the decidua, trophoblastic cells, extracellular matrix, immune cells, and uteroplacental vessels and functions to allow the growth of the embryo while maintaining a regulated blood supply from the mother [32]. The decidua consists of different regions, including the decidua basalis, which is located at the implantation site, and the decidua parietalis, which lines the rest of the uterus. At the implantation site, the decidua basalis and basal plate contribute to a boundary between the placenta and the uterine wall. In normal pregnancies, this boundary serves as a regulatory barrier that prevents excessive trophoblastic invasion into the surrounding uterine tissue. During development of the placenta, trophoblastic cells invade this space; however, they do so in a controlled manner, such that the placenta does not grow too deeply into the uterine wall [33]. When a woman has a previous uterine scar, most commonly after cesarean section or other uterine surgery, the affected area may lose part of its ability to form a proper decidual–myometrial interface. Because of this loss, when the woman becomes pregnant again, her body may be less able to limit the amount of trophoblastic cell invasion into the uterus. This loss of limitation may allow the placenta to attach too deeply or too firmly into the uterine wall, causing severe bleeding and sometimes making delivery impossible without surgical management [34].

Tissue damage due to a previous uterine scar may create a number of ultrastructural abnormalities in the affected area. These abnormalities may make the damaged area more susceptible to additional injury caused by trophoblastic cell invasion. Collagen fibers may become disorganized, leading to decreased elasticity and strength of the damaged area. Fibroblasts may lose part of their ability to produce new collagen and other components necessary for healthy tissue repair. Chronic inflammation may cause continued remodeling of collagen and ECM. Continued activity within the damaged area may disrupt how it responds to physical stress [35]. Trophoblastic cells, being able to sense the physical properties of their surroundings through integrins, may adjust their growth pattern based on what they perceive. Abnormal matrix signaling may therefore contribute to deeper invasion into the uterine muscle. Mechanistically, this may involve integrin-mediated mechanotransduction, focal adhesion kinase, YAP/TAZ signaling, β-catenin stabilization, cytoskeletal remodeling, and invasion-related transcriptional programs [36]. These pathways should be interpreted as biologically plausible associations rather than universally proven causal mechanisms in PAS. A second way in which the maternal–fetal interface may fail to regulate trophoblastic invasion is through inadequate decidualization. Decidualization is the progesterone-dependent transformation of the endometrial stroma into a specialized maternal tissue layer. It involves both transcriptional activation and suppression of various genes involved in proliferation, invasion, stromal maturation, and immune response. Properly regulated decidualization helps prevent uncontrolled trophoblastic invasion. When decidualization fails, inappropriate regulation may contribute to excessive trophoblastic invasion and PAS-like abnormal placentation. A number of factors have been identified as contributing to defective decidualization, including HOXA10, HAND2, WNT4, and progesterone receptor coactivator defects; epigenetic remodeling defects; fibronectin degradation; laminin disorganization; hyaluronan imbalance; increased matrix metalloproteinase activity; and reduced vascular density [37]. Further complications may arise from abnormal vascular structure in the scar site. Decreased vascular density reduces local oxygen availability and impairs diffusion of oxygen across the implantation zone. Reduced vascular density may result in prolonged activity of hypoxia-inducible pathways, including HIF-1α and HIF-2α. Prolonged activity of HIF-1α and HIF-2α may sustain vascular endothelial growth factor (VEGF), glucose transporter protein 1 (GLUT1), carbonic anhydrase IX (CAIX), glycolytic enzymes, angiogenic mediators, and epithelial-to-mesenchymal transition-related genes [38].

In addition to vascular abnormalities, pseudohypoxic signaling may contribute to the pathogenesis of PAS. Pseudohypoxia refers to conditions in which cells activate low-oxygen adaptive programs even when oxygen availability is not uniformly reduced. Adaptations to low oxygen may include glycolytic reprogramming, lactate accumulation, local acidification, redox imbalance, and proteolytic degradation of the ECM. Proteolytic degradation and oxidative injury to ECM components may further weaken the decidual–myometrial boundary. Single-cell RNA sequencing data provide additional support for the view that PAS is associated with disruption of multiple pathways involved in maternal–fetal interface regulation. PAS is characterized by abnormal developmental trajectories in trophoblastic cells, including increases in invasive extravillous trophoblast populations and dysregulated syncytiotrophoblast differentiation, as well as altered expression of TWIST1, SNAIL, ZEB1, and TGF-β signaling molecules [39].

Together, these results suggest that PAS consists of sustained states of invasive behavior among trophoblastic cells within abnormal environments created by decidual remodeling and scarring. Additionally, the decidual immune environment is reorganized through changes in uterine natural killer (NK)-cell receptor profiles, macrophage polarization toward pro-remodeling phenotypes, reductions in regulatory T-cell populations, and increases in pro-inflammatory chemokine signaling. These changes may undermine immune restriction of trophoblastic cell invasion while inducing endothelial stress and pro-inflammatory activation. Furthermore, biomechanical instability may exacerbate these processes. The decidua has sufficient elastic properties to absorb forces generated by placental growth and uterine contractions. However, scar-related decidual implantation regions may display reduced elasticity, abnormal tensile stress distribution, and localized mechanical instability [40]. Additionally, computer-based biomechanical models suggest that these abnormalities may disrupt microvascular structure, increase local ischemic vulnerability, and compromise the structural integrity of the maternal–fetal boundary [41]. From this viewpoint, PAS can be envisioned as a progressively unstable invasive ecosystem based upon defective decidualization, disruption of the ECM, abnormal mechanotransduction, hypoxic signaling, immune remodeling, vascular maladaptation, and altered tissue mechanics. From this viewpoint, invasive placentation represents not just an isolated defect in placental adhesion, but a progressively destabilizing biological condition that may reduce maternal structural resilience before clinical decompensation occurs. Table 1 presents a summary of molecular, biomechanical, inflammatory, and vascular anomalies that ultimately create conditions at the maternal–fetal interface that become chronically invasive and structurally weak in PAS.

### 2.2. Angiogenic Disequilibrium, Endothelial Stress Fields, and the Emergence of Vascular Fragility Architecture

The establishment of a functional placental vascular network through transformation of the spiral arteries represents one of the most significant vascular remodeling events in human biology. The development of this vascular bed involves collaboration among many different cellular components, including endothelial cells, vascular smooth muscle cells (VSMCs), pericytes, and the ECM scaffold. Through their interactions, these components regulate angiogenic signaling, vessel maturation, permeability, and vascular sensitivity, thus facilitating placental exchange [29].

However, when invasive placentation occurs, it disrupts typical vascular formation. The available evidence suggests that there is an imbalance in angiogenic regulation so that some areas of the placental vasculature develop irregular branching geometry, incomplete participation of VSMCs, inappropriate endothelial signaling, irregular microvascular arrangement, and decreased hemodynamic stability [52]. Instead of developing adaptive vascular structures for blood transport across the placenta, many of these networks may form mechanically weak circulatory territories that could become vulnerable during placental separation, uterine contractions, or hemorrhage. One of the primary contributors to the mechanical instability of these vascular systems is dysregulation of the VEGF/VEGFR signal transduction pathway. Disproportionate or spatially irregular activation of VEGF-A, PlGF, VEGFR-1, and/or VEGFR-2 may promote excessive growth of new blood vessels; however, it may also hinder endothelial maturation and stabilization. Elevated angiopoietin-2 may also inhibit protective Ang-1/Tie2 signaling, thereby activating endothelial cells, promoting glycocalyx loss, increasing capillary leakage, attracting leukocytes, and reducing vascular quiescence [53]. Therefore, PAS-associated vascular beds may exhibit diminished elastic content, attenuated contractile responses, irregular branching morphology, and diminished ability to withstand acute increases in blood pressure or flow during hemorrhage [54].

In addition to disrupting normal angiogenic regulation and producing mechanically unstable vascular networks, invasive placentation may also lead to hemodynamic heterogeneity. Abnormally remodeled placental vasculature may produce regions exposed to oscillatory shear stress, rapid fluctuations in blood flow, and turbulence [55]. Since endothelial cells are highly responsive to these mechanical stimuli, long-term exposure to oscillatory shear stress may activate NF-κB signaling, increase mitochondrial reactive oxygen species (ROS) production, diminish nitric oxide (NO) availability through impaired endothelial nitric oxide synthase (eNOS) activity, and cause vasomotor endothelial dysfunction [56].

Another major mechanism contributing to vascular destabilization is oxidative injury. Oxidative stress caused by mitochondrial ROS can initiate lipid peroxidation and DNA damage, disrupt mitochondrial membranes, and induce redox-dependent inflammatory signaling, such as NLRP3 inflammasome and NF-κB activation [57].

Loss of glycocalyx integrity, involving components such as syndecan-1, heparan sulfate, and hyaluronic acid, can additionally compromise both endothelial barrier function and mechanosensing. Reduced NO bioavailability can further exacerbate vasodilatory failure and endothelial exhaustion. There is growing evidence that ferroptosis-related mechanisms may contribute to vascular fragility in invasive placentation. Iron dysregulation, glutathione deficiency, GPX4 dysfunction, and phospholipid hydroperoxide accumulation may compromise the structural integrity of endothelial membranes and diminish their response to hemorrhagic stress [58].

Given that placental tissues are metabolically active and involved in iron homeostasis, they may be especially susceptible to chronic redox imbalance and ferroptotic sensitization. While ferroptosis in PAS-associated vascular failure remains a hypothetical area of study requiring further clinical verification, it is evident that vascular destabilization does not need to occur solely at the implantation site. Placenta-derived extracellular vesicles (EVs) containing inflammatory mediators, oxidized lipids, metabolic regulators, and procoagulant factors could facilitate endothelial activation and oxidative stress signaling throughout the maternal circulation [59].

High levels of placenta-derived microparticles have been linked to maternal endothelial dysfunction and microvascular instability in severe placental disease. Additionally, high levels of IL-1β, IL-6, TNF-α, and chemokines may foster low-grade inflammation during pregnancy and enhance maternal vascular vulnerability to hemorrhagic stress [60].

Concurrently, activated endothelium combined with invasive trophoblastic tissue factor (TF) may stimulate thrombin generation and platelet activation. Activation of platelets by TF may deplete hemostatic reserves, contribute to systemic endothelial dysfunction, and promote diffuse microvascular impairment [61]. Maternal energy utilization may be altered due to placental hypoxia, inflammation, oxidative stress, endothelial dysfunction, and mitochondrial stress. These alterations may affect glucose metabolism, fatty-acid oxidation, oxidative phosphorylation, ROS generation, and endothelial NO signaling, and therefore may increase susceptibility to redox injury and ferroptosis-like processes within the maternal vascular system [62,63].

Therefore, invasive placentation can establish a biological architecture of vascular fragility through dysregulation of angiogenesis, endothelial mechanobiology, oxidative signaling, coagulation, inflammation, mitochondrial function, and microvascular hemodynamics. Consequently, catastrophic hemorrhage can be viewed not only as a mechanical disruption of individual vessels, but also as a manifestation of progressive vascular destabilization that develops over gestation [40].

### 2.3. Placental Invasion as a Chronic Systems-Level Destabilizing Engine

Evidence is growing that invasive placentation can create biological effects outside the maternal–fetal interface. Ongoing interaction of trophoblastic cells with maternal vessels may continuously release various forms of placenta-generated biological signals into the maternal circulation, such as mitochondrial fragments, cell-free DNA, oxidized lipids, syncytiotrophoblast-derived extracellular vesicles, damage-associated molecular patterns, and proangiogenic molecules over an extended period of time [29]. These signals could potentially disrupt the normal functioning of the maternal endothelium, macrophages, coagulation pathways, oxidative-stress responses, and inflammatory processes in maternal vascular beds distant from areas of invasion [59].

It has become increasingly apparent that invasive placentation creates significant immunological alterations during pregnancy. PAS and severe placental disease have been associated with chronic low-grade maternal inflammation characterized by elevated levels of IL-1β, IL-6, TNF-α, chemokines, complement activation, and inflammasome signaling, which may lead to inflammatory cytokine release and subsequent endothelial dysfunction and injury within maternal vascular tissues [64]. Activation of NLRP3 inflammasome signaling may cause further endothelial injury due to IL-1β-induced inflammation and pyroptotic cell-death pathways. Furthermore, activation of the complement system can induce membrane attack complex formation, glycocalyx degradation, platelet activation, and microvascular thrombogenicity. In addition to causing immune-mediated vascular injury, invasive placentation may contribute to maternal coagulopathy through sustained endothelial activation and TF-mediated thrombin generation. Prolonged stimulation of TF expression and activation of coagulative pathways may result in platelet depletion, endothelial exhaustion, and depletion of maternal hemostatic reserves [65]. Simultaneously, placental hypoxia, oxidative stress, inflammatory signaling, and endothelial dysfunction may result in decreased oxygen-use efficiency and mitochondrial adaptability while increasing energy demand [66]. Additionally, inflammatory mitochondrial stress may also contribute to systemic metabolic reprogramming, including altered glucose utilization, reduced fatty-acid oxidation, diminished oxidative phosphorylation, and increased ROS production. Such changes may diminish endothelial NO signaling while increasing ferroptosis-related susceptibility across maternal vascular beds [63].

Therefore, collectively, these studies provide support for the hypothesis that invasive placentation functions as a chronically activated systems-level destabilizing mechanism that may progressively disturb vascular, inflammatory, endothelial, coagulative, metabolic, and bioenergetic homeostasis during gestation [67]. From this viewpoint, EPH may be viewed not only as a therapeutic intervention in response to uncontrolled hemorrhage, but also as a clinical indicator of progressive erosion of maternal resilience via interconnected disruptions in endothelial function, inflammation, coagulation, microvascular flow, metabolism, and mitochondrial function [40].

## 3. Maternal Hemodynamic Resilience and the Exhaustion of Compensatory Reserve

### 3.1. Pregnancy-Induced Cardiovascular Remodeling and the Physics of Hemodynamic Resilience

The primary physiological alterations occurring in the pregnant female cardiovascular system are related to the degree of circulatory remodeling that occurs to satisfy the increased metabolic and perfusion requirements of pregnancy. Total circulating blood volume increases, flow is redistributed from organ to organ within the body, vascular compliance is changed, the ability of the endothelium to respond to mechanical stimuli is altered, mitochondria are recalibrated in terms of their oxidative functions, and oxygen transfer across the maternal vascular bed is adjusted based on changing maternal metabolic demands [68]. The fact that maternal circulation is dynamic and dependent upon flow characteristics indicates that it can be viewed both as a hydraulic system and as an integrated biological network regulated by chemical, rheological, metabolic, and physical processes [69].

Nitric oxide (NO), produced by nitric oxide synthase (NOS), plays a critical role in gestational vasodilation through relaxin and prostacyclin signaling and endothelin suppression. Additionally, NO has been implicated in erythrocyte deformability, endothelial Ca^2+^ signaling, capillary recruitment, maintenance of mitochondrial redox balance, and synchronization of microvascular function under conditions of high hemodynamic stress [70,71].

Given these roles, it appears likely that NO may help integrate both macrovascular and microvascular adaptations during pregnancy. Increased maternal blood volume and cardiac output alter shear stress throughout the circulation, influencing laminar and oscillatory flow patterns [72]. Generally, laminar shear stress promotes endothelial quiescence by activating Krüppel-like factor 2 (KLF2), stimulating eNOS phosphorylation, and inhibiting nuclear factor-kappa B (NF-κB) signaling. However, vascular branching abnormalities, abnormal vascular compliance, and disordered neovascularization that occur in association with invasive placentation may create areas of oscillatory shear stress, which have been found to disrupt endothelial structure and signaling. Although this type of stress may contribute to PAS-associated maternal vulnerability, this hypothesis still requires validation in obstetric populations [73].

Endothelial cells are capable of sensing the mechanical forces induced by flow through the conversion of these mechanical signals into inflammatory, metabolic, and transcriptional events. This process is thought to involve deformation of the glycocalyx, signaling through the PECAM-1 complex, activation of Piezo1 channels, and cytoskeletal remodeling [74]. If oscillatory shear stress continues over time, endothelial mitochondrial production of reactive oxygen species (ROS) may increase, homeostasis of NO may be disrupted, fragmentation of the glycocalyx may occur, and the endothelium may adopt an activated phenotype. It is possible that such changes may contribute to unstable uteroplacental vascular tone prior to overt hemorrhage; however, at present, this hypothesis should be considered biologically plausible rather than proven in EPH patients [75,76].

Arterial remodeling supports the required increase in cardiac output needed to provide adequate perfusion to the fetus and mother. Simultaneously, remodeling of venous capacitance vessels helps to maintain preload reserve during abrupt circulatory disturbances. Compliance of venous capacitance vessels is influenced by autonomic signaling, elastic properties of the ECM, endothelial responsiveness, and energy utilization by vascular smooth muscle cells [77]. Inflammation may compromise this reserve by promoting collagen crosslinking, elastin degradation, and matrix metalloproteinase activity. Consequently, the capacity of veins to compensate for rapid decreases in intravascular volume caused by hemorrhage may be reduced [78]. In general terms, these mechanisms may help explain why some women remain apparently stable at the level of macrohemodynamics despite having extensive placental pathology, whereas other women experience rapid deterioration when subjected to hemorrhagic stress [79]. The fundamental concept is that these molecular pathways do not individually cause collapse, but may instead reduce cardiovascular reserve and decrease the maternal ability to adapt. Future obstetric research is necessary to evaluate how these modifications in endothelial cells, rheology, venous capacitance vessels, and mitochondrial function influence PAS severity, hemorrhage tolerance, and the necessity for emergency peripartum hysterectomy [80].

Similarly, significant adaptations also occur within the heart. Examples include improved mitochondrial function, modified calcium handling, metabolic flexibility mediated by AMPK/sirtuin signaling activation, regulation of mitochondrial fission–fusion dynamics, and PGC-1α-dependent biogenesis [81]. However, if exposure to inflammatory cytokines and catecholamines persists with concomitant endothelial dysfunction, decreased stability of mitochondrial membranes and reduced oxidative phosphorylation activity may limit further cardiac energetic reserve during hemorrhagic shock [82].

Regulation of oxygen transport is greatly affected by maternal physiology. Oxygen transport requires both overall circulatory flow and erythrocyte deformability, heterogeneity in capillary transit times, mitochondrial oxygen extraction, and geometric properties affecting oxygen diffusion in complex microvascular networks [83]. Initial increases in 2,3-BPG concentrations and recruitment of additional capillary beds assist in enhancing oxygen delivery to tissues functioning near their hypoxic threshold. However, eventual impairment of erythrocyte deformability during extreme obstetric stress through oxidative damage to erythrocyte membranes, inflammatory acidosis, and lipid peroxidation can prevent adequate oxygen transfer to tissues, even when total oxygen delivery appears globally sufficient [84].

Important studies demonstrate that endothelial mitochondria are crucial in regulating vascular adaptation. While endothelial cells produce ATP primarily through glycolysis, endothelial mitochondria play important roles in regulating nitric oxide levels, calcium-wave propagation, redox status, inflammasome activation, and mechanosensitive vascular coordination [85]. Mitochondrial dysregulation and dysfunction may generate large-scale hemodynamic stress fields that progressively diminish whole-circulatory resilience during invasive placentation [86].

Therefore, we propose that maternal hemodynamic resilience is maintained by ensuring multiscale circulatory coherence through integrated actions of endothelial mechanotransduction, flow rheology, mitochondrial energetics, oxygen transport efficacy, venous compliance, and metabolic synchrony across vascular territories. Therefore, maternal collapse may result not only from bleeding itself, but also from an inability to sustain this adaptive circulatory architecture under severe obstetric stress [87].

**Figure 1:**
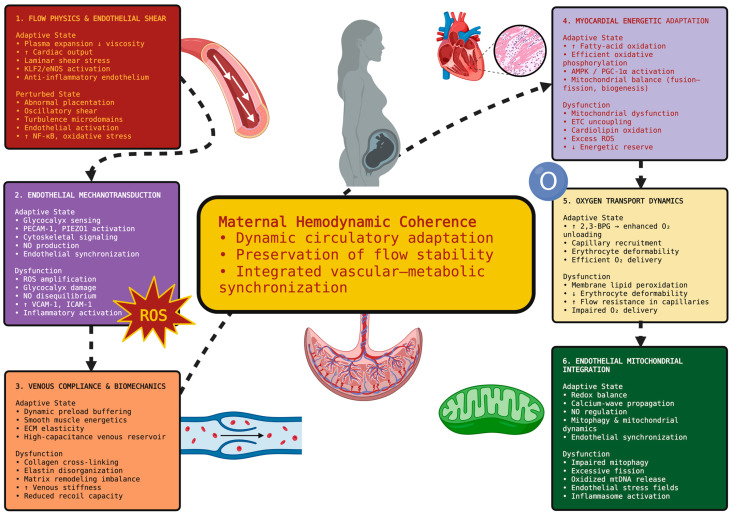
Maternal cardiovascular adaptation during pregnancy is depicted as an integrated multiscale hemodynamic network centered on preservation of circulatory coherence. Physiologic adaptation involves coordinated regulation of laminar flow dynamics, endothelial mechanotransduction, venous compliance, myocardial energetic remodeling, oxygen transport efficiency, and endothelial mitochondrial signaling. Created in BioRender. Stoica, E.-E. (2026). https://app.biorender.com/illustrations/6a1062041e834c7feeef7a11.

Figure 1 illustrates the multiscale adaptive systems required to maintain maternal hemodynamic resilience throughout pregnancy. Figure 1 also illustrates how flow physics, endothelial mechanotransduction, venous compliance, myocardial energetics, oxygen transport dynamics, and mitochondrial signaling function cooperatively to preserve circulatory coherence under increasing physiological loads.

### 3.2. Microcirculatory Decoherence, Endothelial Entropy, and the Hidden Physiology of Compensated Instability

Numerous reports indicate that many patients experiencing severe obstetric complications may have seemingly normal macrocirculatory parameters while having significant cellular and microvascular dysfunction [88]. Patients can appear to have normal systolic blood pressure and cardiac output while displaying signs of endothelial injury, mitochondrial dysfunction, impaired oxygen diffusion, and capillary shutdown. These examples illustrate that macrohemodynamic measures may not accurately reflect microhemodynamic dysfunction, suggesting that catastrophic collapse may occur after extended periods of physiological instability with reduced microvascular coherence despite stable systemic circulation [89].

A self-regulated adaptive exchange network for the distribution of oxygen, nutrients, inflammatory mediators, and metabolic substrates across various tissue territories defines the microcirculation. Under resting conditions, synchronized perfusion and oxygen extraction across the microcirculation are achieved through coordinated communication and signaling between endothelial cells, involving endothelial oscillations, NO gradients, ATP-mediated purinergic signaling, and Ca^2+^ waves [90]. Therefore, stability in microvascular circulation is determined not only by perfusion pressure, but also by coordinated communication and signaling among endothelial cells [91].

Degradation of the glycocalyx due to severe obstetric stress may mark the beginning of microvascular destabilization. The glycocalyx serves as a nanoscale mechanosensory interface controlling shear transduction, barrier integrity, leukocyte migration, and colloid osmotic equilibrium [92]. Degradation of syndecan-1, heparan sulfates, and glypicans by oxidative stress, pro-inflammatory proteases, complement activation, and matrix metalloproteases can cause mechanosensory failure, endothelial dysfunction, breakdown of permeability barriers, interstitial edema, and increased distance for oxygen diffusion. Even relatively small changes in oxygen diffusion distance may significantly impair oxygen transfer efficiency in tissues operating close to hypoxic thresholds [93].

As the glycocalyx degrades, other concurrent events, including endothelial cell activation and swelling, platelet adhesion, leukocyte adhesion, fibrin micromatrix formation, and plasma viscoelasticity changes, contribute to non-homogeneous transit times across microvessels. Areas of the microcirculation may develop functional shunt-like behavior, permitting rapid transit of well-oxygenated blood and preventing maximal extraction in those areas, while other regions receive inadequate perfusion. Thus, hypoxia can develop without proportional decreases in total oxygen delivery [94,95].

Mitochondrial dysfunction can also promote this process. Decreased ATP production in endothelial cells disrupts cytoskeletal organization, junctional integrity, NO production, and ion balance. Activation of inflammasomes such as NLRP3 and cGAS–STING signaling by mitochondrial ROS additionally contributes to generalized inflammation, disruptions in endothelial permeability, and widespread microvascular injury [96]. In summary, these pathological events define the conceptually descriptive term “endothelial entropy,” signifying loss of microvascular synchrony, unstable oxygen diffusion gradients, reduced energetic coordination, and increased spatial heterogeneity in tissue perfusion despite apparently normal systemic hemodynamics [97].

Disequilibrium in nitric oxide, redox imbalance, calcium instability, and functional collapse in mitochondrial bioenergetics may continue to degrade physiological vasomotion, leading to chaotic oscillatory behavior analogous to phase decoherence in nonlinear systems approaching critical transitions [98,99].

Consequently, catastrophic collapse in maternal circulation may be preceded by a long “hidden” period of compensated instability characterized by “endothelial entropy,” “microvascular decoherence,” “mitochondrial energetic failure,” and “diffusion failure,” while systemic circulation appears clinically intact [100].

### 3.3. Nonlinear Hemodynamic Collapse, Critical Threshold Dynamics, and the Thermodynamics of Adaptive Exhaustion

Catastrophic collapse in maternal circulation does not always occur as a simple linear consequence of hemorrhage alone. Rather, collapse may occur when cumulative obstetric stress exceeds the maternal body’s capacity to adaptively maintain circulatory organization [101,102]. Throughout pregnancy, maternal physiological functions operate at extremely high degrees of complexity, requiring substantial amounts of energy to maintain homeostasis and inhibit excessive inflammatory responses. With each advancing week of gestation, maternal physiological reserves are subjected to repeated stressors, including increased inflammation, oxidative stress, endothelial injury, and energy expenditures required to maintain vascular compliance and tissue oxygenation [103].

Continued energy expenditure is required to maintain vascular organization, including preservation of endothelial barrier integrity, ion gradients, stable oxygen diffusion efficiency, and coordinated hemodynamic signaling. As previously discussed, increases in oxidative damage, mitochondrial calcium overload, endothelial injury, glycocalyx degradation, and sustained sympathetic stimulation can accelerate system-wide destabilization [104,105].

When physiological systems approach their limits, physiological behavior tends to become nonlinear. Each successive episode of hemorrhage, placental abruption, reperfusion injury, inflammation, amniotomy, or anesthesia-induced vasodilation can act as a “transition trigger,” rapidly destabilizing previously compensating hemodynamic systems [92]. Chaos can develop within microvascular flow patterns, communication between endothelial cells can fail, oxygen extraction can drop below minimal requirements, and mitochondrial ATP production can fail to maintain organized vascular function. Additionally, energy failure at the tissue level can occur before clinical manifestations of systemic hypotension or shock. It follows that conventional monitoring methods may underestimate actual physiological instability until macrohemodynamic failure becomes clinically apparent. At this point, system-wide resilience may be substantially compromised [106,107].

Flow within microvessels becomes increasingly disordered. Oxygen extraction fails. Coordination among endothelial cells fails. ATP generated by mitochondria becomes insufficient for ordered vascular function. Therefore, when tissue-level energy failure occurs before clinical evidence of systemic hypotension, often referred to as “hidden shock”, a potentially hazardous time interval may exist before standard monitoring accurately reflects the severity of underlying instability. By the time clinical manifestations indicative of systemic hemodynamic compromise are apparent, maternal system-wide resilience may already be severely diminished [108].

Therefore, the concept of “adaptive failure” may offer a conceptual framework for describing mechanisms contributing to catastrophic maternal collapse. We propose that maternal physiological reserves may initially compensate through continued expenditure of available endothelial, respiratory, autonomic, mitochondrial, metabolic, and rheological reserves. Once cumulative energy expenditure exceeds available physiological reserves, however, the maternal circulation may enter an unstable state with positive feedback loops involving oxidative injury, endothelial damage, inflammatory spread, coagulopathy formation, mitochondrial collapse, and diffusive failure [109]. Emergency hysterectomy performed for obstetric hemorrhage may therefore constitute not only a surgical procedure for managing hemorrhage, but also clinical evidence of multiscale adaptive failure occurring within molecular, endothelial, microvascular, and systems-physiology domains. Catastrophic maternal collapse may therefore not result from a single insult alone, but rather from convergence upon a critical transition state at which biological order becomes energetically unsustainable [110].

## 4. Immune–Endothelial–Coagulative Amplification Cascades and the Self-Propagation of Catastrophic Maternal Destabilization

### 4.1. Endothelial Glycocalyx Disintegration, Mechanobiologic Failure, and the Collapse of Immunovascular Homeostasis

The maternal vascular endothelium is a highly mechanically responsive interface where all elements of maternal cardiovascular physiology converge to provide integrated vascular responses and ensure maternal cardiovascular balance. Due to increased blood volume, increased cardiac output, alterations in shear force, vascular remodeling, angiogenesis, and the constant demand of the placenta for perfusion, the vascular endothelium is subjected to continuous high biomechanical stress during pregnancy [111]. To preserve its function and continue to support maternal cardiovascular health, the vascular endothelium must maintain intact structural and functional adaptations. These include preserved glycocalyx structure, functioning nitric oxide signaling, flexibility in endothelial cell shape and cytoskeletal arrangement, appropriate responses to mitochondrial-generated signals, and a functional mitochondrial redox state. When catastrophic obstetric stress occurs, these adaptations may fail, resulting in endothelial dysfunction and transforming the endothelium from a protective vascular interface into a contributor to maternal cardiovascular destabilization [112].

One key area of failure is the glycocalyx. The glycocalyx consists of syndecans, glypicans, heparan sulfate proteoglycans, hyaluronate chains, and membrane-bound glycoproteins. The glycocalyx serves as a nanoscale mechanochemical sensor of fluid flow over the endothelium, controlling activation of nitric oxide synthase, paracellular barrier function, leukocyte interaction with the vessel wall, and vascular rheology [113]. Oxidative stress can disrupt glycocalyx structure by inducing MMPs to cleave syndecans and heparan sulfates, promoting neutrophil elastase release, and inducing inflammatory cytokines that cleave various glycocalyx components. Collectively, these mechanisms can profoundly alter the physical characteristics of blood flow adjacent to the endothelium [114]. Reduced glycocalyx thickness alters the laminar flow pattern near the endothelium and increases oscillatory shear forces acting on the endothelial surface. Reduced NO production through impaired eNOS function, combined with altered mechanotransduction involving YAP/TAZ signaling and enhanced NF-κB-mediated inflammatory gene expression, may result in disturbed vasomotor tone and increased variability of capillary flow. Additionally, disruptions in VE-cadherin junctions, claudins, and occludin-containing tight junction complexes may increase vascular permeability by changing capillary diffusion geometry, thus decreasing the efficiency of oxygen delivery [115].

Mitochondrial function in endothelial cells plays a critical role in many of these processes. Despite being predominantly glycolytic, endothelial mitochondria are essential for calcium sequestration, regulation of ROS production through modulation of respiration, regulation of inflammasomes, and activation of mechanotransductive pathways. Continuous placental inflammatory stimulation and oxidative stress may stimulate DRP1-dependent mitochondrial fission, increase cardiolipin oxidation, and decrease respiratory chain efficiency. Mitochondrial fission may increase mitochondrial ROS production and impair mitophagy. Oxidized mtDNA has been shown to activate cGAS–STING and NLRP3 inflammasome pathways. These pathways are thought to further destabilize endothelial function [116].

In addition to oxidative stress-induced damage, mechanical deformation causes cytoskeletal rearrangements in endothelial cells, including actin stress-fiber formation, focal adhesion disruption, and increased endothelial cell stiffness, thereby decreasing vascular viscoelasticity. Increased mechanical load transmitted to fragile microvessels by rigid endothelial cells may result in further vascular injury [117].

Endothelial cells also communicate through ATP-based purinergic signaling, Ca^2+^ wave propagation, extracellular vesicle transfer, oxidatively modified phospholipids, inflammatory miRNAs, mitochondrial-derived fragments, and placenta-derived procoagulant signals. Hence, catastrophic maternal destabilization may be accompanied by a gradual loss of mechanobiochemical coherence throughout the endothelial system before obvious clinical manifestations of hemorrhagic decompensation appear [118].

### 4.2. Immunothrombotic Network Reorganization, Ferrocoagulative Instability, and the Collapse of Hemostatic Adaptation

The maternal coagulation system during pregnancy is regulated in a restrictive yet adaptive manner. On the one hand, it prevents excessive hemorrhage while maintaining flow in the microcirculation. Hemostasis is distributed throughout the immunovascular network as an end-to-end connection between endothelial cell signaling, platelet interactions with other platelets and endothelial cells, inflammation, fibrinolysis, complement activity, mitochondrial redox signaling, and flow rheology [119]. Severe hemorrhage or placental insufficiency can transform this adaptively organized system into a self-reinforcing thromboinflammatory process. This transformation may amplify endothelial damage, reduce tissue perfusion through inadequate oxygen delivery, and increase oxidative stress. Ultimately, this may contribute to dysfunction of the maternal circulation and cardiovascular system [120].

Neutrophil extracellular traps (NETs) seem to play an important role in the transition from a hemostatically or adaptively organized maternal coagulation system into a maladaptive immunothrombotic system. Neutrophils undergoing NETosis release chromatin NETs that consist of extracellular DNA, citrullinated histones, myeloperoxidase, neutrophil elastase, and granule-derived proteases. The presence of chromatin NETs near the vessel wall can significantly affect blood rheology by creating areas of turbulence near the vessel wall through altered viscoelastic properties of blood, altered laminar flow profiles, and creation of turbulent microdomains within small-diameter capillaries [121]. Citrullinated histone proteins released from NETs are harmful to endothelial cells because they can cause membrane instability, increased intracellular calcium levels, and mitochondrial dysfunction. NET-derived fibrin networks may also exhibit greater branch density, smaller pore sizes, larger elastic modulus values, and reduced susceptibility to plasmin-mediated lysis compared with non-NET-derived fibrin networks [122].

Platelets are also involved in this transformation. Upon activation, platelets produce numerous agonists, including serotonin, thromboxane A2, calcium ions, polyphosphates, ATP, inflammatory cytokines, and phosphatidylserine-enriched vesicles that bind to platelet surfaces. These agonists support tissue-factor expression and activation of procoagulant activity, in addition to enhancing platelet activation and thromboinflammation [123].

Studies have demonstrated that complement systems contribute to destabilizing maternal immunothrombotic states. Activation of C3a and C5a through classical or alternative pathways leads to recruitment of neutrophils into damaged vascular beds. C5a activation enhances endothelial permeability and increases oxidative stress production by neutrophils. C5a activation also stimulates tissue-factor expression in damaged vascular beds. Deposition of membrane attack complex (MAC) onto endothelial membranes disrupts membrane integrity and glycocalyx structure. Interaction between MAC deposited on platelet surfaces and activated platelets generates positive feedback loops that enhance NETosis and microvascular thrombogenicity [124].

Important studies have suggested that ferrocoagulative mechanisms may also contribute to catastrophic coagulative failure. Massive hemorrhage or hemolysis results in large amounts of iron entering the circulation. Transfusion exposure or oxidative stress generated by endothelial injury may release even more catalytically active iron into the circulation. Catalytically active iron reacts with H_2_O_2_ produced by NADPH oxidase-derived superoxide through Fenton and Haber–Weiss reactions, producing hydroxyl radicals. Hydroxyl radicals rapidly oxidize phospholipids contained in the outer leaflet of endothelial cell plasma membranes and platelet membranes. Oxidized phosphatidylethanolamines and lipid aldehydes may stimulate tissue-factor expression, induce mitochondrial dysfunction, propagate inflammatory responses, and damage membrane structure in endothelial cells throughout vascular beds [125].

Additionally, fibrinolysis is spatially heterogeneous. Early hyperfibrinolysis may develop due to elevated tPA activity stimulated by activated endothelium; however, local fibrinolytic shutdown may develop due to elevated PAI-1 or thrombin-activatable fibrinolysis inhibitor (TAFI) activity in some vascular beds, resulting in simultaneous hemorrhage and microvascular thrombosis in different vascular territories. Consequently, some vascular beds may exist under hemorrhagic conditions while others experience microvascular thrombosis, ultimately leading to widespread disruption in hemostatic coherence [126].

Systems physiology describes catastrophic maternal coagulopathy as a nonlinear transition from adaptive hemostatic organization toward chaotic immunovascular propagation. Thrombi limit oxygen diffusion into tissues, causing mitochondrial hypoxia. Hypoxia increases oxidative stress and continues to damage the endothelium, resulting in persistent stimulation of coagulative activation that may consume maternal hemostatic capacity in a self-amplifying fashion [127]. Table 2 lists several mechanisms that combine to drive catastrophic maternal coagulopathy into a self-amplifying state of immunovascular instability.

### 4.3. Cytokine Resonance, Pyroinflammatory Synchronization, and Systems-Level Propagation of Catastrophic Collapse

Catastrophic maternal deterioration should not be interpreted merely as an increase in cytokine concentration when inflammation during severe obstetric stress is considered as a systems phenomenon. Rather, increased cytokine concentrations during catastrophic maternal decompensation may help generate a self-sustained inflammatory state similar to synchronized instability seen in nonlinear coupled systems [138].

Multiple pathways mediate inflammatory responses, including IL-1β, IL-6, TNF-α, HMGB1, interferons, and related mediators. These regulate endothelial barrier function, mitochondrial energy production, calcium signaling, coagulation, and cellular energy organization through multiple cross-talking signaling pathways, including JAK/STAT, NF-κB, MAPK, and inflammasome pathways. Under normal circumstances, these regulatory mechanisms are limited by counter-regulatory actions provided by anti-inflammatory cytokines, mitochondrial redox regulation, metabolic checks, and suppressive immune cells. However, during catastrophic hemorrhage or placental compromise, these regulatory mechanisms may progressively fail [139].

The NLRP3 inflammasome represents a potential biological mechanism linking mitochondrial damage with both endothelial activation and amplified inflammatory responses. A variety of stress signals may contribute to activation of the NLRP3 inflammasome, including accumulation of ROS, exposure of oxidized cardiolipin, extracellular ATP release, cellular potassium ion efflux, and cytosolic exposure of mitochondrial DNA. Once activated, subsequent caspase-1 activation leads to cleavage and processing of pro-IL-1β into mature IL-1β, which may then promote pyroptosis through gasdermin-D activation. This process can result in cell lysis and release of additional DAMPs that may propagate further inflammatory stimulation in adjacent vascular territories [140]. Additionally, extracellular ATP has been shown to increase purinergic signaling through P2X7 receptors in endothelial cells, leading to increased endothelial permeability, calcium-wave propagation, and leukocyte recruitment. Mitochondrial debris released as a result of mitochondrial dysfunction may additionally activate cGAS–STING-dependent signaling pathways as well as Toll-like receptor-dependent signaling pathways in neighboring immune and endothelial cells, resulting in downstream inflammatory responses. Therefore, inflammasome activation could contribute to the propagation of vascular inflammation among interconnected cellular networks, although its direct role in EPH-related vascular collapse has yet to be confirmed clinically [141].

Extreme inflammatory responses may also disrupt intracellular organizational phase space. Oxidative stress and ATP depletion can disrupt membraneless organelle structures, disorder stress-granule dynamics, disrupt protein-folding homeostasis, and impair translational regulation. Disruption of cellular organizational phase space decreases adaptive capability as physiological stress escalates [142].

This can be described using systems-physics concepts as resonance amplification. Cytokine signaling, endothelial damage, mitochondrial dysfunction, coagulation activation, calcium dysregulation, and oxidative stress may work synergistically to produce positive feedback loops. As this synergy grows, progressively smaller local disturbances may produce larger-scale systems effects [143].

Moreover, synchronization among components of the inflammatory cascade may occur before apparent cardiovascular collapse. Decreased coherence among systems responsible for maintaining endothelial integrity, immune responses, coagulation, mitochondrial function, and metabolic homeostasis may occur even while systemic hemodynamic parameters remain unchanged. Therefore, conventional monitoring techniques may underestimate the degree of systems-level collapse occurring while apparently stable clinical reserves remain [144].

Therefore, maternal deterioration may be conceptualized here as pyroinflammatory synchronization, characterized by progressive loss of autonomous regulation among systems responsible for maintaining vascular integrity, immune responses, coagulation, mitochondrial function, and metabolic homeostasis, resulting in self-amplifying inflammatory resonance states [145]. Therefore, emergency peripartum hysterectomy may represent not only a surgical intervention to control bleeding, but also evidence of failure to coordinate regulation among these systems at a macroscopic level [146].

## 5. Bioenergetic Failure, Ferroptotic Destabilization, and the Metabolic Architecture of Irreversible Maternal Collapse

### 5.1. Mitochondrial Network Failure and the Exhaustion of Maternal Bioadaptive Reserve

Increasingly, catastrophic maternal decline may be viewed less as a purely hemodynamic event and more as an evolving failure of distributed bioenergetic coordination. As pregnancy represents one of the greatest physiological energy loads imposed on adult human biology, high demands are placed on mitochondrial adaptation across multiple cell types, including endothelial cells, cardiomyocytes, immune cells, uterine smooth muscle cells, renal tissue, hepatocytes, and placental trophoblasts. These cells require continuous adaptation in order to support oxygen distribution, vascular compliance, inflammatory tolerance, and metabolic flexibility under elevated systemic load [147,148]. Therefore, maternal resilience relies not only on maintaining circulatory reserve, but also on preserving mitochondrial adaptability that can synchronize ATP production, calcium-buffering capacity, redox homeostasis, and inflammatory regulation across multiple organ systems simultaneously [149].

Under normal physiological conditions, mitochondrial resilience is preserved by coordinated regulation of oxidative phosphorylation efficiency, dynamic changes in fusion–fission processes within the mitochondrial network, mitophagy-mediated removal of damaged mitochondria, maintenance of cardiolipin structure and function, balance of the NAD^+^/NADH ratio, and PGC-1α-mediated mitochondrial biogenesis [150]. However, severe placental stress may destabilize each of these systems through chronic endothelial oxidative signaling, endothelial inflammation, ischemia–reperfusion injury, and amplified inflammatory cytokine signaling. Specifically, electron transport chain dysfunction, particularly involving complexes I and III, may result in increased superoxide leakage and disruption of respiratory supercomplex formation due to cardiolipin oxidation [151]. In addition, loss of inner membrane stability decreases the proton gradient necessary for ATP production and may enhance cytochrome c release and opening of the permeability transition pore [152].

Elevated levels of intracellular Ca^2+^ could disturb mitochondrial homeostasis. Excess Ca^2+^, if it exceeds the cell’s adaptive capacity, may cause mitochondrial respiratory activity to become less efficiently coupled to ATP synthesis. There may also be increased ROS generation, in addition to changes in mitochondrial dynamics, including an increased rate of DRP1-mediated fission [153]. As such, a fragmented mitochondrial phenotype is generally associated with inefficient energy metabolism, decreased buffering capacity for cellular stress, impaired elimination of dysfunctional mitochondria through mitophagy, and larger amounts of mitochondrial-derived danger signals being released into the cytosol and extracellular space. These signals may include oxidized mitochondrial DNA, cardiolipin-derived lipid fragments, and N-formyl peptides. As soon as these mitochondrial damage-associated molecular patterns are released into the cytoplasm or extracellular space, they can serve as inflammatory promoters. These mitochondrial DAMPs can trigger inflammation by activating both cGAS–STING pathways and NLRP3 inflammasomes, thereby amplifying innate immune responses [154]. Therefore, mitochondrial fragmentation may represent a way of converting metabolic disturbances into inflammatory amplification mechanisms. While this is a biologically plausible process that may contribute to some of the pathology observed during EPH, additional obstetric research is required before its direct clinical relevance can be validated [155].

It is important to note that catastrophic obstetric stress may cause “cytopathic hypoxia,” a condition in which mitochondrial oxygen utilization is inadequate despite relatively preserved systemic oxygen supply. Nitric oxide disequilibrium, ROS-mediated inhibition of iron–sulfur enzyme activity, inhibition of pyruvate dehydrogenase, and impairment of TCA cycle flux may lead to progressive decoupling of available oxygen from ATP production. Thus, tissues may experience significant energetic collapse before the onset of clinical evidence of gross macrovascular failure [156].

As such, mitochondrial dysfunction may destabilize multiple adaptive domains required for maternal physiological resilience. Energetic deficiency in the endothelium results in impaired glycocalyx turnover, NO synthesis, barrier integrity, and cytoskeletal compliance. Impaired energy status in cardiomyocytes impairs efficient calcium handling and reduces contractile reserve. Impairment of uterine smooth muscle bioenergetics may diminish responsiveness during periods of severe hemorrhage. Metabolic reprogramming of immune cells can lead to enhanced cytokine release, inflammasome activation, and oxidative propagation [157].

### 5.2. Ferroptotic Amplification, Iron-Catalyzed Redox Instability, and Membrane Catastrophe

In recent years, several mechanisms of critical illness pathophysiology have received increasing attention, among which ferroptosis may be particularly relevant for catastrophic obstetric collapse. Catastrophic obstetric collapse can lead to massive bleeding, causing a series of pathological responses that include loss of iron regulation, increased oxidative chemistry, loss of endothelial barrier function, decreased coagulative adaptability, and increased inflammation. These responses are linked to each other and may create a self-reinforcing feedback loop that contributes to catastrophic outcomes [158].

Ferroptosis represents a regulated form of oxidative cell death dependent on iron. It is primarily caused by phospholipid peroxidation and loss of glutathione-dependent membrane antioxidant defense. Massive obstetric hemorrhage may create multiple factors that allow ferroptosis-related processes to propagate. Hemolysis, ischemia–reperfusion injury, exposure to iron from blood transfusions, and oxidative endothelial damage may significantly increase the amount of available catalytic iron in the circulation [159]. Additionally, this free iron provides substrates for Fenton and Haber–Weiss reactions. These reactions produce hydroxyl radicals, which can initiate lipid peroxide formation in mitochondrial, endothelial, erythrocyte, and platelet membranes. Specifically, polyunsaturated phosphatidylethanolamines rich in arachidonic and adrenic acid are particularly susceptible to oxidation via lipoxygenase-mediated pathways [160].

Under normal physiological conditions, glutathione peroxidase 4 (GPX4) detoxifies lipid hydroperoxides produced during lipid peroxidation, thereby preventing membrane damage. However, under catastrophic maternal stress conditions, glutathione may be depleted due to reduced NADPH availability related to impaired electron transport chain activity, mitochondrial dysfunction, inflammation-associated metabolic changes, and impaired cystine transport [160]. As a result of these processes, the ability of GPX4 to protect against oxidative membrane damage is significantly reduced. Therefore, once glutathione levels are sufficiently low, oxidized phospholipids may accumulate rapidly. This may result in disruption of membrane fluidity, ion-channel structure and function, mitochondrial permeability, and the structural integrity of endothelial junctions [161].

However, the effects of ferroptosis-related injury do not end when individual cells die. Oxidized phospholipids can stimulate Toll-like receptor activation, enhance inflammasome signaling, increase tissue-factor expression, and contribute to the development of inflammatory endothelial phenotypes. In addition, lipid aldehydes such as 4-hydroxynonenal and malondialdehyde can bind covalently to various proteins, including mitochondrial enzymes, cytoskeletal components, ion channels, and proteins involved in maintaining endothelial junction integrity. Each modification may reduce the remaining functional reserve of these proteins, thereby contributing to declining vascular coherence [162].

Placental tissue may also exhibit particular susceptibility to ferroptotic destabilization. Placental tissues contain large numbers of mitochondria and experience high rates of oxidative metabolism, as well as substantial iron exchange during gestation. There is growing evidence indicating altered GPX4 activity, ACSL4 upregulation, disrupted iron handling, and accumulated lipid peroxides in disorders of invasive placentation. Collectively, these data suggest that chronic ferroptotic sensitization may occur before catastrophic hemorrhagic collapse occurs during pregnancy [66,163].

Ultimately, ferroptosis-related injury may substantially alter the physical properties of microvessels responsible for delivering nutrients and oxygen to tissues. Specifically, damaged endothelial membranes lose electrochemical stability and mechanosensitive response capabilities, increasing microvascular permeability and disrupting NO-mediated vasodilation. Oxidative modification of erythrocyte membranes impairs deformability, increases capillary resistance, and alters oxygen diffusivity. Finally, lipid oxidation of platelets may contribute to coagulopathy through dysfunctional activation and microparticle formation [164].

**Figure 2:**
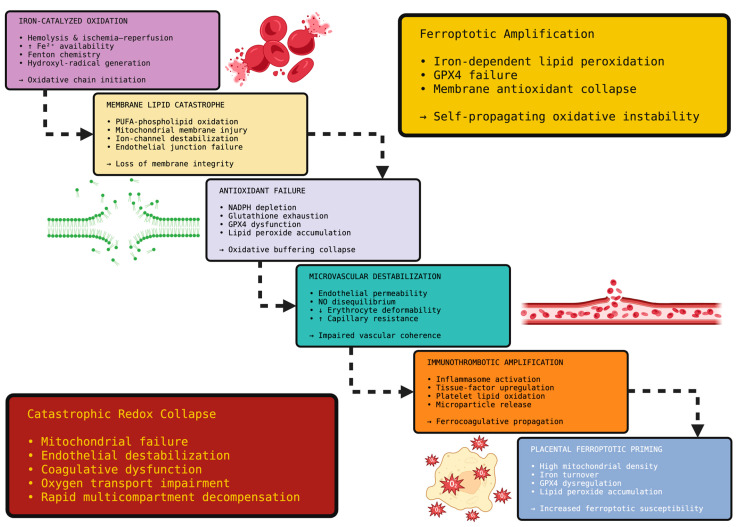
Ferroptotic propagation during catastrophic obstetric collapse is depicted as a coupled oxidative–vascular destabilization cascade initiated by iron-dependent radical generation and phospholipid peroxidation. Created in BioRender. Stoica, E.-E. (2026). https://app.biorender.com/illustrations/6a1095319174bc7d52c6e3eb.

Therefore, catastrophic maternal collapse may be considered a condition of “ferrocoagulative redox propagation”, in which iron chemistry, lipid peroxidation, mitochondrial damage, endothelial injury, and immunothrombotic activation become integrated into a single self-amplifying process. When antioxidant-buffer capacity is overwhelmed, subsequent redox propagation may exhibit characteristics similar to autocatalytic chain reactions found in unstable chemical systems, thereby creating rapid progression of tissue destabilization across multiple vascular territories [165]. This cascade is summarized schematically in Figure 2, highlighting how ferroptosis-related redox imbalance may connect molecular membrane injury with progressive vascular and hemostatic destabilization during catastrophic obstetric collapse.

### 5.3. Metabolic Phase Transition, Energetic Entropy, and the Irreversibility Threshold of Maternal Collapse

Maternal physiological change from compensatory mechanisms toward catastrophic failure may resemble a phase transition in energetic system coordination. During normal pregnancy, physiological processes undergo constant remodeling of glycolysis, oxidative phosphorylation, fatty-acid oxidation, lactate shuttle function, amino-acid metabolism, oxygen sensing, and mitochondrial biogenesis so that energy is supplied to tissues as demand varies [109].

However, when a mother experiences extreme placental, inflammatory, or hemorrhagic stress, her ability to adapt to changing conditions may begin to break down. Mitochondrial ATP production may become less efficient, as indicated by increased reliance on HIF-1α-stimulated glycolysis. Lactate levels may increase due to impaired pyruvate dehydrogenase activity, as well as disruptions in NADH oxidation, TCA cycle flux, and mitochondrial respiratory coupling [166]. ATP loss disrupts many important regulatory processes, including ion-gradient establishment and maintenance, intracellular calcium regulation, cell membrane polarization, and intracellular phase separation. In addition to serving as an energetic molecule, ATP maintains protein structural organization and supports the physical properties of membraneless organelles [167]. Thus, it is not only ATP depletion that disrupts granules involved in stress responses, RNA compartmentalization, ribosomal function, and signaling pathways, but also the loss of ATP-dependent structural support. Increasing oxidative damage, ionic imbalance, and decreased ability of cells to maintain their internal phase organization ultimately reduce the ability of tissues to coordinate and respond to increasing physiological strain [168].

As the body’s energetic systems continue to degenerate, they appear to accumulate “entropy,” similar to what occurs at the systems level during catastrophic collapse of physiological order. Maintaining physiological order requires continued energy expenditure to support vascular endothelial synchrony, mitochondrial communication, calcium-wave propagation, oxygen transport efficiency, and microvascular synchrony. When oxidative damage, inflammation, and mitochondrial dysfunction progress, the amount of energy required to maintain increasingly disordered biology continues to rise. At some point, the cost of maintaining these energetic requirements may exceed the body’s bioadaptive capacity [169].

Systems collapse is characterized by nonlinear threshold behavior similar to critical-state phenomena observed in physics. Once a system has passed its resilience threshold, additional minor disturbances, such as continuing bleeding, anesthesia-induced vasodilation, ischemia–reperfusion injury, further inflammation, or coagulative disruption, may cause disproportionate systemic collapse. Oxygen utilization efficiency decreases dramatically, endothelial synchrony breaks down, mitochondrial ATP production becomes insufficient, and inflammatory spread may accelerate through tightly synchronized feedback loops involving ROS amplification, calcium dysregulation, and cytokine activation [170].

Moreover, extensive metabolic destabilization may occur before evident hypotension or circulatory collapse. Significant mitochondrial dysfunction, endothelial energetic failure, and collapse of tissue oxygen utilization can occur even though traditional hemodynamic data may indicate minimal impairment. Therefore, catastrophic maternal deterioration may represent a prolonged hidden phase of energetic entropy accumulation before collapse becomes evident, during which conventional clinical manifestations may be absent or nonspecific [171].

Consequently, the notion of a “bioenergetic point of no return” may provide a conceptual basis for understanding the mechanisms behind emergency peripartum hysterectomy. EPH may not represent solely a surgical reaction to uncontrollable hemorrhage, but rather a terminal manifestation of simultaneous destabilization of mitochondrial, endothelial, inflammatory, coagulative, and metabolic systems, leading to energetic loss of synchronization and eventual self-amplifying collapse into disorder [172]. Once a system loses energetic coherence, it may no longer be fully restored through external intervention despite vigorous treatment. Catastrophic deterioration may then progress despite intensive medical intervention. Collapse toward an irreversibly disordered state, representing loss of organized physiological order, may occur when the energetic cost necessary to restore such organization becomes biologically insurmountable [11].

## 6. Critical Transition Dynamics, Predictive Instability Signatures, and the Computational Physiology of the Maternal Point of No Return

### 6.1. Catastrophic Obstetric Collapse as a Critical-State Transition

Although catastrophic maternal collapse has historically been considered an acute linear hemorrhagic process, more recent evidence and systems-level interpretations suggest that catastrophic maternal collapse may behave less like an acute linear hemorrhagic event and more like a critical-state transition in a highly connected physiological network. In this framework, each patient may be able to transiently maintain overall physiological stability despite extensive endothelial injury, invasive placentation, inflammatory responses, and significant mitochondrial and microvascular damage, only for the patient’s condition to degrade rapidly after relatively small additional insults, such as placental avulsion, anesthetic-induced vasodilation, reperfusion injury, or minor increases in bleeding [173]. These nonlinear changes suggest that severe obstetric collapse is likely to result from loss of systems-level resilience rather than simply from a proportionate response to the amount of blood lost during delivery [174].

Complex adaptive systems approaching instability, in fields ranging from physics and climatology to neurology and biological systems, typically exhibit a gradual decrease in resilience before catastrophic failure. This pre-collapse phase is characterized by increasing variability, unstable oscillations, decreased ability to recover from disturbances, increased autocorrelation, loss of synchronization, and reduced adaptability, collectively known as critical slowing down. Similar behaviors may also be relevant to catastrophic maternal physiological deterioration [175].

Pregnancy is a unique, high-load, far-from-equilibrium biological state that requires constant synchronization of endothelial mechanotransduction, mitochondrial energy production, clot formation and resolution, inflammatory regulation, oxygen diffusion, autonomic modulation, and metabolic flexibility [176]. Stability is not derived only from the intactness of each system individually, but also from synchronized communication between systems. However, when subjected to extreme stressors caused by severe placental dysfunction and hemorrhage, oxidative glycocalyx fragmentation, mitochondrial dysfunction, enhanced inflammatory responses, heterogeneous rheology, and desynchronized endothelial function may contribute to increasing physiological entropy while decreasing the maternal organism’s ability to absorb additional insults [177].

Therefore, catastrophic collapse can be viewed as a failure of distributed connectivity between multiple adaptive biological systems. Normal physiological resilience is dependent upon redundant pathways, system modularity, distributed compensatory signals, and inhibitory feedback mechanisms that buffer positive feedback loops. Severe obstetric stress may ultimately destabilize these normal protective mechanisms. Eventually, positive feedback loops involving ROS amplification, endothelial injury, mitochondrial fragmentation, ferroptosis, calcium dysregulation, and cytokine signaling may overwhelm compensatory regulatory mechanisms, resulting in highly interconnected instability states prone to abrupt transition into new systemic states [178].

Physical principles also support this view. The maternal circulatory system operates as a dissipative thermodynamic system requiring continuous ATP utilization to maintain organized endothelial signaling, efficient oxygen diffusion, synchronized microvascular function, and metabolic organization under increasing gestational load. With increasing oxidative stress and inflammatory activation, progressively greater amounts of energy may be required to maintain decreasing levels of physiological organization. When the energy required to maintain coherence exceeds the bioadaptive capacity of the system, a sudden transition toward large-scale disorganization may occur, similar to transitions observed in physical systems approaching critical thresholds [179].

Consequently, the “maternal point of no return” may not be a single hemodynamic threshold. Rather, it may represent a critical transitional state in which coordinated maternal biological organization can no longer be restored despite emergency interventions. Therefore, emergency peripartum hysterectomy may represent not only a surgical intervention for hemorrhage control, but also a final manifestation of gradually progressing systems-level destabilization occurring over time throughout gestation across endothelial, mitochondrial, inflammatory, coagulative, and metabolic systems [180].

### 6.2. Hidden Instability Signatures, Physiologic Noise, and the Early Detection of Resilience Failure

If catastrophic maternal collapse behaves as a “critical state”, detectable instability patterns may appear before clinical manifestation of complete decompensation. Evidence continues to grow from nonlinear physiological studies showing that biological systems often exhibit identifiable changes in signal complexity, entropy, oscillatory coherence, recovery characteristics, and physiological variability before total failure occurs. Obstetric catastrophes may present similar opportunities for identifying signs of resilience erosion before the development of systems-level instability that may lead to irreversible maternal deterioration [181].

Conventional monitoring in obstetrics has primarily focused on measurement of static parameters, such as blood pressure, hemoglobin concentration, coagulation factor levels, urinary output, and estimates of blood loss. Complex adaptive systems, however, typically begin to fail through changes in dynamic behavior rather than through absolute deviations in static parameters [182]. For example, heart rate variability (HRV) can illustrate the principle that changes in dynamic behavior may provide early warning signs of impending failure. HRV reflects the normal fractal organization of physiological cardiovascular oscillations resulting from multiscale autonomic integration. As inflammation progresses, endothelial function is compromised, mitochondrial function begins to fail, and the autonomic nervous system becomes exhausted; both the variability and fractal nature of HRV may decline and become rigidified. Paradoxically, this reduction in HRV indicates the reduced ability of the mother to respond adaptively to changing physiological demands [183].

Similarly, microvascular physiology may demonstrate analogous dynamics. Normally functioning endothelial networks exhibit synchronized oscillatory vasoconstriction and vasodilation that maximize capillary recruitment and optimize oxygen delivery throughout heterogeneous tissue territories. Glycocalyx degradation, disrupted intracellular calcium stores, amplified reactive oxygen species production, and mitochondrial dysfunction all contribute to disruption in the dynamic behavior of oscillating microvascular flows. These disruptions may produce unstable topological flow structures and heterogeneous transit times with decreased efficiency of oxygen diffusion before clinically evident hemodynamic collapse occurs [98].

In addition, biochemical instability signatures may manifest through altered temporal dynamics rather than simply being defined by individual concentration thresholds. Temporal variation in lactate kinetic profiles, angiopoietin-2 fluctuation profiles, extracellular mitochondrial DNA release dynamics, cytokine fluctuation profiles, glycocalyx fragment profiles, oxidative lipid metabolite profiles, or ferroptosis-associated phospholipid profiles may potentially reflect decreasing resilience better than simple static measurements. Additionally, increased autocorrelation and delayed physiological response times after perturbation may indicate declining adaptive flexibility and increasing proximity to critical transition states [184].

In particular, mitochondrial signaling may offer sensitivity in this regard. Oxidative damage to mtDNA, cardiolipin fragmentation, and ATP release dynamics directly reflect destabilization within the energy-generating core of the adaptive system. Since mitochondria serve as integrators of inflammatory signaling, endothelial regulation, intracellular calcium homeostasis, and oxygen use, small-scale changes in oscillatory behavior of mitochondrial function may precede large-scale collapse of the overall adaptive system [185].

Finally, advanced biophysical monitoring technologies further extend this framework. Advanced technologies for measuring tissue oxygen saturation fluctuations, visualizing the endothelial glycocalyx, analyzing pulse-wave complexity of blood pressure signals, creating maps of capillary flow topologies, assessing fluorescent signatures of mitochondrial activity, and evaluating microcirculatory coherence may eventually enable clinicians to identify hidden instability states in real time that were previously undetectable using traditional obstetric monitoring modalities [186].

Therefore, “biological noise,” traditionally viewed as meaningless random fluctuation in biological parameters, should now be interpreted as potentially containing valuable information concerning the architectural structure of biological resilience. Decreases in fractal complexity, increases in oscillator rigidity, variance amplification, breakdown of synchrony, and delays in physiological recovery dynamics all have the potential to act as measurable indicators of impending systemic destabilization in obstetric catastrophes [187].

Prediction of catastrophes during childbirth may thus shift from threshold-based detection methods to continuous assessment of the dynamic architecture of instability in obstetric patients. Clinicians may need not only to recognize hemorrhage at the time of collapse, but also to detect gradual erosion of resilience before the maternal organism reaches the biological transition point beyond which restoration of ordered physiological coordination becomes improbable [188].

### 6.3. Artificial Intelligence, Digital Twin Physiology, and Predictive Modeling of Catastrophic Transition States

Catastrophic obstetric collapse as a nonlinear systems phenomenon represents a fundamental challenge to historical prediction models that use risk factors, individually or combined with other static clinical variables, to predict catastrophic events during pregnancy. In contrast to the predictions made by these models, catastrophe may result when the various processes leading to catastrophic collapse become dynamically coupled through resilience depletion [189]. As each process leading to catastrophe generates new information relative to previous time points, catastrophe may not be adequately predicted using static measures, single time-point assessments, or individual risk measures alone. Instead, catastrophes may require prediction from the combination of multiple physiological processes over time as multidimensional trajectories, necessitating advances in artificial intelligence (AI), multimodal physiological modeling, spatial omics technologies, and computational medicine to improve catastrophic obstetric care [190].

In addition to their demonstrated potential to identify latent physiological patterns not detected by traditional statistical analysis, machine-learning systems may integrate diverse biomarkers from different sources, including endothelial biomarkers, inflammatory oscillations, hemodynamic variability, placental imaging phenotypes, mitochondrial signaling, coagulation dynamics, extracellular vesicles, ferroptotic metabolites, and transfusion trajectories [191]. These integrative systems may allow identification of “hidden” instability states before obvious collapse. Furthermore, instead of simply categorizing patients according to static risk levels, they may enable continuous modeling of how the patient’s overall physiological resilience changes over time [192].

Similar to machine-learning systems, placental imaging is undergoing rapid evolution driven by AI algorithms. Radiomics-based texture mapping, flow-turbulence modeling, vascular topology reconstruction, Doppler signal processing, and elastography all contribute to quantitative measurement of anomalous microarchitectural structure within the placenta, extending beyond simple visual assessment. Examples of measurable characteristics indicative of underlying systems-level vascular instability include chaotic neovascular branching patterns, alterations in vascular fractal dimension, local perfusion heterogeneity, and irregularities in the harmonic content of blood flow [193].

At the same time, researchers are employing spatial transcriptomics and single-cell multiomics techniques to provide previously unattainable molecular detail regarding invasive placentation biology. Future studies combining trophoblast differentiation states, endothelial signaling networks, ferroptotic signatures, immune-cell topologies, mitochondrial stress pathways, and extracellular matrix remodeling patterns could ultimately support the development of high-dimensional molecular instability maps that would allow identification of catastrophic resilience-loss states long before delivery [194].

However, perhaps one of the most impactful future developments in catastrophic obstetrics is the concept of digital twin physiology. Digital twin systems represent computational approximations of individual patients created by ongoing integration of imaging data, molecular data, physiological signals, laboratory trajectories, hemodynamic variability, and treatment response into personalized predictive models [195]. Therefore, such systems may be capable of predicting resilience-depletion trajectories, simulating responses to physiological perturbations, identifying impending transitions across thresholds where catastrophic collapse may become irreversible, and forecasting the likelihood of catastrophic transition before irreversible destabilization occurs [196].

As systems theory concepts suggest, digital twins could serve as computational observational platforms for identifying hidden patterns in physiological organization. Unlike identifying disease only after catastrophic collapse has clearly manifested as a clinical entity, future predictive systems may be able to assess the energetic and organizational distance between compensation and irreversible systems-level disintegration. Thus, the primary goal of catastrophic obstetric care might evolve toward preservation of physiological coherence rather than solely hemorrhage management [197].

Therefore, emergency peripartum hysterectomy could be viewed not merely as an operative outcome, but rather as a systems-level indicator of a point at which recovery of coordinated physiological organization may no longer be possible [198].

The future of catastrophic obstetrics may rely not only on advancing the rate of hemorrhage control or increasing transfusion efficacy, but also on developing methodologies to identify, measure, and potentially reverse early-stage hidden instability states in pregnant patients before patients cross the threshold beyond which restoration of organized physiological behavior becomes biologically improbable [199].

## 7. Precision Rescue Strategies, Systems-Level Therapeutic Modulation, and the Future Reversibility of Catastrophic Maternal Collapse

### 7.1. From Hemorrhage Control to Restoration of Systems-Level Physiologic Coherence

In recent years, there has been a growing body of evidence suggesting that, in many cases, catastrophic maternal deterioration, including shock, may still occur after apparently successful surgery for postpartum hemorrhage (PPH). This may be due to the fact that the primary biological processes associated with this type of event are much broader than circulatory loss alone. These include, but are not limited to, endothelial glycocalyx fragmentation, disruption of mitochondrial activity, disorganization of microcirculatory function, increased ferroptotic stress, immunothrombosis, and inflammatory amplification [200]. In addition to being technically successful, it is therefore possible that a successful “catastrophic obstetric rescue” will require more than restoration of individual physiological variables, such as correction of coagulopathy, and instead require restoration of what might be termed “distributed physiologic coherence” [201].

Increasingly, severe obstetric collapse can be viewed as a destabilized adaptive network operating near energetic and informational failure thresholds. Thus, when vasopressors are used to stabilize the maternal cardiovascular system, it should be recognized that improving systemic blood pressure does not necessarily equate to restoring synchronized endothelial function, efficient oxygen diffusion, mitochondrial ATP production, or proper microvascular flow organization [202]. Furthermore, aggressive use of crystalloids to expand intravascular volume may increase venous return and subsequently cardiac output; however, excessive crystalloid administration can also reduce glycocalyx thickness and contribute to endothelial damage secondary to stretching forces generated by excess intravascular fluid. Excessive use of crystalloids may also decrease colloid osmotic pressure, increase interstitial edema formation, and potentially worsen diffusion distances for nutrient and oxygen delivery to tissues [203].

Therefore, the definition of “hemodynamic success” may need to undergo significant refinement. For example, successful recovery may depend not only on quantitative aspects of flow, such as blood pressure, but also on qualitative characteristics of vascular structure and flow architecture. These structural characteristics include microvascular organization and coherence, intact endothelial mechanoreception and sensing mechanisms, mitochondrial oxygen consumption efficiency, red-cell deformability, and synchronized flow patterns through individual capillaries [204].

Endothelial glycocalyx preservation may be one of the most important biological considerations during acute postpartum hemorrhage resuscitation. Preservation of the endothelial glycocalyx maintains vascular integrity and permeability regulation, as well as shear-stress-regulated nitric oxide release and subsequent smooth-muscle relaxation. Additionally, preservation of the glycocalyx promotes leukocyte exclusion mechanisms, limits inflammation, and protects against mechanical and electrochemical forces that disrupt normal flow dynamics [205]. Therefore, plasma-rich resuscitation protocols that maintain balanced fibrinogen levels, albumin-mediated oncotic stabilization, and endothelial-protective transfusion practices may provide benefits that extend beyond coagulation factor replacement. Ultimately, these interventions may promote vascular flow harmony and preserve capillary oxygen-exchange architecture [206].

Preservation of mitochondrial function may be equally critical. Mitochondria support cellular ATP generation and metabolic coordination. During severe hemorrhagic insult and subsequent inflammation, progressive ATP depletion destabilizes multiple endothelial barrier functions, including calcium-wave synchronization, ion-gradient maintenance, cytoskeletal organization, and intracellular phase behavior across multiple organ systems [207]. Emerging experimental data using metabolic supportive therapies, including pyruvate supplementation, NAD^+^ restoration, AMPK activation, mitochondrial membrane stabilization, succinate buffering, and cardiolipin preservation, indicate that catastrophically deranged inflammatory states may be reversible at an energetic level, provided that intervention occurs before irreversible fragmentation of mitochondrial networks [208].

Timing of interventions may play a particularly important role in this context. Complex adaptive systems often have small time windows, or narrow reversibility windows, before collapse, during which restoration of organized function remains biologically feasible. After these time windows have passed, systems destabilization may continue through self-reinforcing feedback loops independent of the initial hemorrhagic stimulus, regardless of how aggressively external correction is attempted [209].

Thus, emergency peripartum hysterectomy may ultimately be viewed not simply as an act of surgical hemorrhage control, but as an effort to interrupt a self-organizing cascade leading to system-wide maternal failure before the point at which restoration of biological coordination becomes biologically unattainable [210].

### 7.2. Endothelial Reprogramming, Ferroptosis Suppression, and Emerging Molecular Rescue Ecosystems

There have been significant advances in recent years in understanding how specific biological mechanisms respond to various forms of maternal decompensation. The study of these mechanisms, including those involving vascular biology, immune response, mitochondrial function, and critical care physiology, provides evidence that maternal decompensation may not always represent an immediately irreversible condition. Rather, maternal decompensation appears to be an evolving and potentially treatable biological process until it reaches a state of complete loss of physiologic homeostasis [211].

One key mechanism that may play a role in potential therapies for catastrophic obstetric collapse involves endothelial stability. The angiopoietin-1/Tie2 signaling pathway is essential for maintaining endothelial quiescence, preserving structural and functional integrity of the glycocalyx, regulating tight junctions between cells, and promoting vascular efficiency [212]. Excessive angiopoietin-2 release disrupts Tie2-mediated signaling pathways, leading to increased endothelial permeability, glycocalyx degradation, inflammatory adhesion-molecule expression, and microvascular leakage in severe obstetric stress. Experimental use of Tie2 agonists and angiopoietin-2 inhibitors has demonstrated marked endothelial-protective effects in inflammation associated with critical illness. Therefore, it is possible that future rescue treatments for catastrophic obstetric collapse may involve direct endothelial reprogramming and not merely correction of coagulopathy [213].

Another potential strategy for treating acute hemorrhagic shock is modulation of nitric oxide–ROS coupling. Depletion of tetrahydrobiopterin (BH4) due to oxidative damage disrupts endothelial nitric oxide synthase (eNOS) function, shifting nitric oxide-producing pathways toward superoxide-generating pathways that promote oxidative damage and endothelial disorder. Restoring endothelial redox balance through antioxidant buffering, BH4 protection, regulation of mitochondrial ROS production, or conservation of NADPH may potentially help restore coordinated endothelial function and capillary integrity under extreme hemorrhagic conditions [214].

Suppression of ferroptosis provides another innovative approach to treating catastrophic obstetric collapse. Significant research indicates that iron-dependent lipid peroxidation creates a connection between hemorrhage, mitochondrial failure, endothelial injury, destabilization of coagulation cascades, and inflammatory amplification within a single self-sustaining redox network [215]. Early intervention strategies may include preservation of GPX4 function, replenishment of glutathione levels, iron chelation, inhibition of lipoxygenases, and elimination of reactive phospholipid peroxides. Additionally, mitochondria-targeted antioxidants capable of protecting cardiolipin structure and preserving respiratory supercomplex organization may help prevent endothelial energetic collapse and inflammatory synchronization [216].

Regulation of inflammasomes presents another potential treatment strategy. Activation of NLRP3 integrates mitochondrial ROS production with extracellular ATP release, cellular Ca^2+^ dysregulation, recognition of oxidized lipids, and ferroptotic injury into pyroinflammatory amplification. Inhibiting inflammasome assembly, gasdermin-mediated pyroptosis, purinergic signaling, and extracellular mtDNA recognition may therefore attenuate systems-level inflammatory synchrony and endothelial fragmentation during catastrophic maternal stress [217].

Finally, regulation of NETosis represents another emerging frontier. Excessive formation of neutrophil extracellular traps (NETs) can significantly alter microvascular rheology, fibrin ultrastructure, endothelial permeability, and capillary oxygen diffusion. Potential therapeutic approaches may include agents designed to degrade NET chromatin and histones, regulate complement activation, and block immunothrombotic pathways, thereby restoring more normal microvascular fluid dynamics and attenuating diffuse endothelial stress propagation [218].

It should be noted that future therapeutic designs for catastrophic obstetric collapse will likely shift toward precision molecular rescue ecosystems rather than isolated treatments. Endothelial nanotherapeutics, targeted exosome therapy, artificial intelligence-guided metabolic optimization, mitochondrial substrate-delivery systems, biomaterials for glycocalyx repair, and adaptive transfusion algorithms may ultimately act in concert to sustain physiological coherence across multiple organ systems during catastrophic obstetric instability [219].

Collectively, these emerging paradigms suggest that biological reversal of catastrophic maternal decompensation is determined not only by the extent of hemorrhage, but also by whether endothelial synchrony, mitochondrial organization, and metabolic coherence can be restored before these networks become irreversibly fragmented [220].

### 7.3. Precision Obstetrics, Digital Twin Physiology, and the Prevention of the Maternal Point of No Return

Catastrophic obstetric collapse is increasingly recognized as an abrupt nonlinear transition in maternal systems-level dynamics. Future goals of maternal health will therefore need to incorporate preventive strategies that continually assess the dynamic architecture of resilience across simultaneous systems of endothelial function, mitochondrial function, inflammation, coagulation, metabolism, and hemodynamics [221].

Obstetrics is moving toward the development of multidimensional physiological maps capable of recognizing hidden instability states before overt clinical failure becomes evident. Using data provided by placental radiomics, vascular fractal analysis, Doppler flow harmonics, extracellular vesicle profiling, glycocalyx biomarkers, mitochondrial metabolites, ferroptotic phospholipids, and inflammatory oscillations, it may become possible to determine how systems-level adaptive reserves decline during pregnancy [222].

What is important here is that the way in which systems behave over time may provide biological information. Decreasing physiological complexity, loss of physiological rhythmicity, oscillatory rigidity, delayed recovery of physiological function after perturbation, autonomic nervous system desynchronization, capillary flow heterogeneity, and increased noise in endothelial function may each represent measurable indicators of decreasing resilience that occur before traditional laboratory abnormalities appear. Thus, catastrophic obstetric monitoring is evolving away from static threshold-based detection of instability toward continuous assessment of dynamic stability architecture [223].

Because catastrophic transitions occur due to interactions among many poorly coupled physiological variables, with behavior that may remain undetectable using traditional statistical approaches, artificial intelligence systems are uniquely positioned to help address this problem. A machine-learning algorithm incorporating biomarkers of endothelial function, mitochondrial signaling, coagulation oscillation dynamics, inflammatory trajectory patterns, radiomic placental patterns, and physiological variability signatures could potentially detect latent instability states that precede overt collapse by hours or even days [224].

A further advancement within this area of study may include the development of digital twin physiology. Digital twins are defined as continuously updated computational representations of individual patients that integrate molecular data, images, physiological signals, laboratory trajectories, microvascular function, and treatment responses into dynamic predictive models. Using this approach, theoretical models of catastrophic obstetrics could potentially predict resilience-depletion trajectories, simulate responses to physiological disturbances, estimate when critical transition thresholds are approaching, and quantify the likelihood of irreversible systems-level physiological destabilization before catastrophic collapse becomes clinically evident [225].

With this conceptual structure in mind, emergency peripartum hysterectomy would not simply represent a surgical endpoint, but could also indicate that the patient’s systems biology has crossed a point where reversible physiological organization may no longer be possible. Therefore, the major task of future maternal medicine may be preservation of biological coherence itself. In the final analysis, the future of catastrophic obstetrics may rest upon the ability to identify the underlying architecture of instability, restore synchrony between endothelial and mitochondrial functions, prevent the spread of ferroptosis and pyroinflammation, maintain energy-based organization, and therapeutically reduce the rate of resilience decline before the mother crosses the biological threshold beyond which restoration of coordinated adaptive order becomes biologically improbable.

## 8. Conclusions

Traditionally, EPH has been viewed primarily as a surgical intervention for life-threatening obstetric complications, such as severe postpartum hemorrhage, placental abruption, placenta accreta spectrum disorders associated with catastrophic bleeding, uterine rupture, or failure of non-surgical hemorrhage-control techniques. However, recent discoveries in endothelial biology, mitochondrial energy metabolism, immunothrombosis, ferroptosis, systems medicine, and computational physiology suggest that emergency peripartum hysterectomy may also occur in the setting of progressive disruption of maternal homeostasis, flexibility, and adaptive coordination during pregnancy.

During pregnancy, which can be considered a high-energy, multiscale biological condition, multiple physiological processes require continuous coordination to support fetal growth and maternal adaptation. These include endothelial cell mechanosensing, mitochondrial ATP synthesis, microvascular blood flow, clotting mechanisms, inflammatory tolerance, oxygen transport, autonomic nervous system regulation, and metabolic flexibility. Thus, the maternal organism is able to respond appropriately to changing conditions by allowing these systems to work together under increased mechanical and metabolic loads placed on the uterus, placenta, and maternal vasculature throughout gestation. Maternal stability therefore depends upon maintaining proper coordination among systems working synergistically. Disruption of communication among these systems may progressively decrease maternal resilience and increase vulnerability to catastrophic decompensation.

The disruption associated with placenta accreta spectrum disorders may begin locally at the site of abnormal placental invasion into the myometrium. However, PASDs may also be associated with broader alterations in physiological systems involved in endothelial mechanotransduction, mitochondrial ATP production, coagulative organization, inflammatory signaling, metabolic adaptability, glycocalyx maintenance, nitric oxide regulation, ferroptotic lipid peroxidation, NETosis, inflammasome activation, extracellular-vesicle signaling, and microvascular blood-flow distribution. Each of these physiological systems may communicate with other systems through feedback mechanisms, allowing them to interact as a network of interdependent physiological processes.

Thus, disruption in the functioning of one component of this network may contribute to disruption in other components, resulting in a cascade effect that increases instability of the entire network. When this happens, some women with PASD may appear clinically stable until they suddenly decompensate and develop catastrophic outcomes. This may occur because traditional measures used to assess maternal well-being, including blood pressure, pulse oximetry, cardiac output, and standard laboratory values, may appear normal even while underlying physiological disturbances are already progressing. This concept suggests that maternal collapse may occur rapidly and catastrophically as a result of multiple interacting disturbances rather than as a direct consequence of acute blood loss alone. It represents a paradigm shift from understanding maternal collapse as a linear response to acute hemorrhagic trauma toward viewing it as a nonlinear collapse of maternal physiological systems, analogous to a tipping point in a complex adaptive system.

As such, emergency peripartum hysterectomy does not merely serve as a means to rapidly control hemorrhage, but may also represent the end stage of systemic destabilization across multiple physiological domains, including endothelial, mitochondrial, immune, coagulative, microvascular, and metabolic systems.

Therefore, future approaches in obstetric critical care may focus on preserving the coherence of physiological systems responsible for supporting life rather than only managing hemorrhagic shock. Potential strategies may include endothelial repair agents, therapies designed to preserve glycocalyx integrity, ferroptosis inhibitors, mitochondrial-targeted energetic support, anti-thromboinflammatory agents, inflammasome inhibitors, multimodal monitoring devices using machine-learning algorithms and artificial intelligence predictive models, and digital twin-based simulation models designed to identify unstable physiological patterns before clinical collapse occurs. Although this approach remains hypothetical and requires clinical validation, this framework offers a systems-level explanation for catastrophic maternal deterioration. Ultimately, EPH may represent the final clinical evidence of a maternal system that has lost the capacity to maintain coordinated biological coherence.

## Figures and Tables

**Table 1:** Major molecular, immunologic, metabolic, extracellular matrix, vascular, and biomechanical alterations that collectively transform cesarean scar implantation zones into chronically invasion-permissive microenvironments in the placenta accreta spectrum.

Pathobiological Layer	Core Dysregulation	Key Molecular/Biophysical Drivers	Functional Shift	Tissue-Level Consequence	References
Decidual barrier failure	Incomplete stromal differentiation	HOXA10, HAND2, WNT4, NOTCH, FOXO1, progesterone coactivators	Reduced decidual resistance	Excess trophoblast penetration	[42]
Scar niche remodeling	Chronic dysregulated tissue repair	Collagen disorganization, elastin loss, ECM turnover imbalance	Viscoelastic instability	Persistent invasion-permissive interface	[43]
Mechanotransduction activation	Abnormal stiffness and tension gradients	Integrins, FAK, YAP/TAZ, β-catenin, cytoskeletal remodeling	Sustained invasive signaling	Trophoblast persistence beyond physiological limits	[44]
ECM destabilization	Structural matrix fragmentation	Fibronectin cleavage, laminin disarray, hyaluronan imbalance, MMP activation	Reduced interface integrity	Weak decidual–myometrial separation	[45]
Hypoxia-associated persistence	Chronic low-oxygen signaling	HIF-1α, HIF-2α, VEGF, GLUT1, CAIX	Maintained survival and migration programs	Pseudohypoxic invasive phenotype	[46]
Metabolic reprogramming	Shift toward glycolytic dependence	Lactate accumulation, redox imbalance, glycolytic enzymes	Acidic invasive microenvironment	Proteolytic ECM injury and oxidative stress	[47]
Aberrant trophoblast trajectories	Persistence of immature invasive states	TWIST1, SNAIL, ZEB1, TGF-β signaling	Stabilized EMT-like invasion	Expansion of EVT-like subpopulations	[48]
Immune niche remodeling	Loss of invasion-restrictive immune balance	Uterine NK-cell receptor alteration, macrophage polarization, Treg imbalance, chemokines	Pro-remodeling inflammatory state	Reduced immune-mediated containment	[49]
Vascular maladaptation	Impaired microcirculatory architecture	Reduced vessel density, endothelial stress, ischemic remodeling	Oxygen-diffusion instability	Sustained hypoxic signaling	[50]
Biomechanical fragility	Abnormal force distribution and stress concentration	Altered anisotropy, tensile imbalance, reduced tissue resilience	Amplified mechanical instability	Progressive tissue destabilization	[33]
Integrated invasive ecosystem	Convergence of hypoxia, inflammation, ECM failure, and altered mechanics	Coupled molecular–biophysical feedback loops	Chronic invasion-permissive state	Progressive PAS evolution	[51]

**Table 2:** Integrates the principal interacting drivers of catastrophic obstetric coagulopathy, emphasizing how NET-associated thrombogenesis, platelet inflammatory signaling, complement activation, ferroptotic lipid injury, endothelial destabilization, and spatially heterogeneous fibrinolysis become progressively synchronized into a self-propagating immunovascular state characterized by microthrombotic obstruction, oxidative amplification, and loss of hemostatic stability.

Instability Driver	Principal Molecular/Cellular Trigger	Systems-Level Effect	Resulting Structural/Functional Outcome	References
NET-driven immunothrombosis	Release of extracellular DNA, citrullinated histones, myeloperoxidase, and elastase during NETosis	Disruption of blood-flow rheology and amplification of thromboinflammatory signaling	Capillary flow disturbance, endothelial injury, and formation of rigid fibrin-rich networks	[21]
NET-associated fibrin restructuring	Incorporation of NET scaffolds into densely branched fibrin matrices	Reduced fibrin accessibility to plasmin and impaired fibrinolytic clearance	Persistence of mechanically resistant microthrombi	[128]
Platelet-mediated inflammatory activation	Release of thromboxane A2, serotonin, ATP, polyphosphates, and extracellular vesicles	Enhancement of tissue-factor activity and propagation of endothelial activation	Amplified coagulative signaling across the vascular network	[129]
Platelet mitochondrial dysfunction	ROS generation and release of inflammatory microparticles from activated platelets	Coupling of thrombotic activity with oxidative stress pathways	Oxidative injury within the microvasculature	[130]
Complement-driven amplification	C3a/C5a signaling and membrane attack complex deposition	Recruitment of neutrophils, increased endothelial permeability, and reinforcement of NETosis	Glycocalyx injury and escalating thrombogenicity	[131]
Iron-dependent redox coagulation	Iron overload and radical generation via Fenton and Haber–Weiss chemistry	Propagation of lipid peroxidation and oxidative membrane injury	Structural instability of endothelial and platelet membranes	[132]
Oxidized phospholipid propagation	Accumulation of oxidized phosphatidylethanolamines and reactive lipid aldehydes	Promotion of tissue-factor signaling and mitochondrial dysfunction	Escalation of inflammatory and coagulative injury	[133]
Spatial fibrinolytic imbalance	Endothelial tPA release combined with simultaneous PAI-1 and TAFI activation	Regional dissociation of fibrinolytic activity	Coexistence of active bleeding and microvascular thrombosis	[134]
Hypoxic microvascular feedback loop	Impaired oxygen diffusion caused by microthrombotic obstruction	Amplification of mitochondrial ROS production	Progressive endothelial dysfunction and worsening perfusion failure	[135]
Endothelial decompensation	Glycocalyx breakdown, calcium overload, and membrane destabilization	Loss of vascular regulatory integrity	Self-propagating coagulative activity	[136]
Nonlinear hemostatic collapse	Synchronization of interacting thromboinflammatory feedback loops	Breakdown of adaptive hemostatic regulation	Self-sustaining immunovascular instability	[137]

## Data Availability

No new data were created or analyzed in this study. Data sharing is not applicable to this article.
